# A Helmet of Her Own?: A Qualitative Study of Key Stakeholder Perspectives on Headgear in Girls’ Lacrosse

**DOI:** 10.1016/j.focus.2023.100078

**Published:** 2023-02-08

**Authors:** Shivani Iyer, Kathleen Bachynski

**Affiliations:** Department of Public Health, Muhlenberg College, Allentown, Pennsylvania

**Keywords:** Girls’ lacrosse, concussion, gender, headgear, injury prevention

## Abstract

**Introduction:**

Despite the high incidence rate of concussions in women's lacrosse, there is substantial push back against the use of helmets at the secondary educational and collegiate levels in the U.S. This study examines the social factors influencing the controversy surrounding the use of protective headgear and how the recent development of headgear specific to the women's game has shaped ongoing debates.

**Methods:**

Purposeful sampling was used to recruit interviewees with research knowledge or firsthand experience with injury and protective headgear in girls’ lacrosse. Semistructured interviews were conducted and subsequently coded using qualitative research software to identify key themes and patterns.

**Results:**

Sixteen respondents participated in this study; 4 players, 4 coaches, 3 researchers, and 5 administrators were represented in this sample. Overarching themes identified across these interviews included playing through pain, health consequences of concussions, concussion prevention strategies and the potential role of headgear, symbolism of headgear, gender dynamics, autonomy, and decision making.

**Conclusions:**

This qualitative study shows the importance of attention to the unique history of girls’ lacrosse and current narratives surrounding headgear in the sport. There is a need for greater collaboration and consensus between all relevant groups to ensure that headgear best addresses the concerns of the people who will ultimately be using it. Future qualitative research should build on this preliminary study with a larger and more diverse sample to follow up on key themes and ultimately inform effective safety measures to protect athletes.

## HIGHLIGHTS

• Girls’ lacrosse has a high incidence of concussions and traumatic brain injury.
• Headgear specific to the game became available in 2017 but remains contentious.
• Gender, autonomy, and risk compensation are key factors informing headgear debates.
• To effectively sustain new safety measures, these social factors must be considered.

## INTRODUCTION

In the U.S., girls’ and women's lacrosse is quickly growing in popularity across age and skill levels, seeing a nearly 54% increase in the number of high school–aged players alone between 2008 and 2018.^1^^,^^2^ This growth has highlighted significant concern over traumatic brain injuries (TBIs) in lacrosse, particularly over the incidence of concussions. During seasons played between 2008 and 2009 and 2018–2019, the national rate of concussions for high school girls’ lacrosse players was 3.91 per 10,000 athletic exposures.^1^ Recent data collected through the National High School Sports-Related Injury Surveillance Study and the National Collegiate Athletic Association Injury Surveillance Program found that concussions accounted for the most injuries reported in high school girls’ lacrosse competitions (35%) as well as 21.2% of all injuries at the college level.^3^ Other studies have confirmed high rates of concussions in girls’ lacrosse, finding that the sport has anywhere between the second to the seventh highest concussion rate of any sport examined at either of these levels.^1^^,^^3^^,^^4^

The history and rules of girls’ lacrosse are separate from those of boys’ lacrosse and inform the most common mechanisms of injury. Girls’ lacrosse developed from a version of the sport played by Native American men; it was introduced to and adapted for girls in Scotland and Britain in the late 19th century.^5^ Subsequently, American physical educators imported the girls’ sport to the U.S. in the early 20th century and founded the U.S. Women's Lacrosse Association in 1931.^5^ Prevailing expectations of femininity at the time included notions that the female body was not physically capable of exerting energy without severely disrupting reproductive ability.^5^ American girls’ lacrosse was established in this context, notably including restrictions on physical contact for women.^5^ The game has since grown to be characterized by rules that promote strategy, agility, and finesse over force and speed.^5^^,^^6^

To this day, in contrast to the boys’ game, girls’ lacrosse is a noncontact sport whose rules prohibit intentional collisions with the body and/or equipment.^5^^,^^6^ Despite these rules, nearly three quarters of concussions in the girls’ game are caused by a lacrosse ball or stick illegally coming in contact with a player's head.^1^^,^^6^^,^^7^ However, in boys’ lacrosse, which is contact heavy by design, athlete–athlete contact accounts for a much greater percentage of concussions (66.4%) than in girls’ lacrosse (19.8%).^1^^,^^6^

To address the risk of head injury in the women's game, an American Society for Testing and Materials International performance standard (F3137) specific to girls’ lacrosse was developed.^7^ This is notable because historically, girls’ lacrosse players have competed with only eye goggles and mouth guards and without the protective headgear or padding mandated in the boys’ game.^1^^,^^5^ Headgear meeting this American Society for Testing and Materials standard typically includes a flexible exterior and covers the top and back of the skull.^7^^,^^8^ It became publicly available for purchase in 2017 and is currently an optional form of equipment under U.S. Lacrosse, National Federation of State High School Associations, and National Collegiate Athletic Association rule books.^7^^,^^8^ Because some school districts, leagues, and most notably the state of Florida began requiring their players to wear headgear, controversy arose among members of the lacrosse community in regard to the purpose and need for headgear.^2^^,^^9^

Although recent research has measured how efficacious helmets can be in preventing concussions, there is an incomplete understanding of the social and cultural factors that influence varying stances on headgear implementation.^2^ This study examines the perspectives of various stakeholders and explores the impact these beliefs have on health behaviors and safety measures to protect female lacrosse players.

## METHODS

### Study Population

In this qualitative study, semistructured, one-on-one interviews with key stakeholders in girls’ lacrosse were conducted from June 2021 to January 2022.^10^ First, a narrative analysis methodology was employed to examine existing written sources pertaining to headgear and safety in girls’ lacrosse.^11^ These included PubMed articles, local and national newspapers, and magazines ranging from the 1980s to the present day. In addition, school board meeting minutes and other written sources were examined from several specific case studies of school districts, institutions, and states that either implemented or removed headgear mandates for their players. From these sources, several themes emerged that helped to identify key stakeholders in the debates and informed the development of interview questions regarding specific attitudes and beliefs surrounding protective equipment.

Purposeful sampling was used to recruit interviewees with particular research knowledge or firsthand experience with injury and protective headgear in girls’ lacrosse.^12^ To ensure that the sample was representative of major stakeholders, interview candidates were categorized into 4 main groups: (1) players, (2) coaches/referees/parents, (3) researchers, and (4) administrators. The names and/or contact information used to reach out to interviewees were obtained either through mention in written sources described earlier or through snowball sampling by verbal reference from a previous interviewee.^13^ Players, parents, and coaches with varying experiences in using and/or implementing headgear at either the high school or college level were recruited. Researchers consisted of experts in the field with at least 1 peer-reviewed publication in the area of head protection or concussions in girls’ lacrosse. Administrators included representatives from local school boards as well as state-level and national governing bodies.

### Measures

Upon receiving approval from the Muhlenberg College IRB, potential participants were contacted through e-mail. Written informed consent, through a Google Form questionnaire, and verbal consent were obtained before the start of each interview. Semistructured interviews, lasting between 30 minutes to 1 and a half hours, were conducted and recorded through the video conferencing platform Zoom. Open-ended interview questions were prepared in advance and included a selection of common questions asked of all interviewees (Table 1). These addressed respondents’ knowledge of and experience with headgear options in the sport, sources of information on injury risks in the sport, the role of rules and style of play in girls’ versus boys’ lacrosse, understandings of decision-making authority regarding lacrosse rules and policies, and attitudes toward whether helmets/headgear should be optional or mandatory in the sport. In addition, questions specific to each interviewee were asked depending on such factors as their role/involvement with the game and experiences with lacrosse injury. Interviews were stored in Panopto, an educational video management system where video and audio recordings of the interviews were tagged and organized on the basis of each participant's role. Automatic transcripts were generated for each interview.

**Table 1:** Sample Interview Questions

Category	Question
Common questions asked in all interviews	1. Can you explain your role and its relationship with the sport of women's lacrosse? 2. How has awareness and prevention of concussions and traumatic brain injuries changed over time during your involvement with girls and women's lacrosse? 3. What are your thoughts on the existing headgear on the market that meet ASTM standard F3137-15? 4. A recurring concept in research on headgear in women's lacrosse is the “gladiator effect.” What is your opinion on this concept? Do you believe that the use of protective headgear will make the sport of lacrosse more or less aggressive? 5. I have heard the concern that adding helmets to lacrosse would make the sport more like the boy's game. Is this consistent with what you have seen and do you believe it to be true? 6. Can you speak to your perspective on concerns related to maintaining the “integrity” of girls’ lacrosse? In your opinion, what does “maintaining integrity” mean? In what ways does protective headgear impact girls’ lacrosse's integrity? 7. Do you feel that implementing/mandating helmets in the sport is enough to reduce the incidence of concussions in the sport? Do you feel that any other efforts are necessary to lower the number of concussions? 8. Who do you believe should be responsible for deciding whether players are required to wear headgear? 9. Do you believe helmets/headgear in women's lacrosse should be mandatory or optional? Why or why not?
Sample follow-up questions tailored to specific interviewees	1. What was your opinion of the FHSAA's decision to mandate helmets in 2015 before the establishment of an ASTM standard/approved helmet design specific to the sport in 2017? Do you feel that this decision prioritized player safety? (This question only asked to stakeholders in administrative/research roles/from the state of Florida who would be familiar with this decision.) 2. As a survivor of a brain injury and advocate for brain injury prevention, can you speak to the ways in which concussions have had long lasting effects on you? What challenges were associated with her recovery? (This question only asked to players with experience of TBI/coaches with children who were injured as only they are able to provide this perspective.)

ASTM, American Society for Testing and Materials; FHSAA, Florida High School Athletic Association; TBI, traumatic brain injury.

### Data Analysis

Transcripts were coded using Dedoose (Version 9.0.18, 2021, SocioCultural Research Consultants; LLC, Los Angeles, CA; www.dedoose.com), a mixed-methods data management and analysis software. The goal of coding was to identify key themes and patterns across interviews. This was achieved using an iterative coding process, where several rounds of coding were conducted by the 2 researchers to adjust and refine the preliminary codebook, with regular conversations and reviews of codes to address any discrepancies. During this process, codes were added, combined, and/or redefined on the basis of new themes that were emerging as interviews were being reviewed. After solidifying the codebook, interviews were coded individually.

## RESULTS

Sixteen interviews were conducted and transcribed between the summer of 2021 and the winter of 2022 and later coded for analysis. Four players, 4 coaches, 3 researchers, and 5 administrators were represented in this sample (Table 2). Of the respondents, 9 women and 7 men participated. There was some overlap in roles held by participants; for example, 1 administrator was also a parent to an athlete, and 2 players served as referees and/or coaches in the summers. Four major themes and a series of subthemes were identified from the interviews (Table 3) and are outlined below.

**Table 2:** Table of Participants, n = 16

Primary role	Additional primary role details	Secondary role(s)
Player (n = 4)	Concussion/TBI survivor Former player	N/A
	Concussion/TBI survivor Current player Does wear headgear	N/A
	Current player Does not wear headgear	Coach/referee
	Concussion/TBI survivor Current player Does wear headgear	Coach/referee
Coach/parent/referee (n = 4)	Youth leagues/high school team coach	N/A
	Parent to concussion/TBI survivor	Coach/referee
	High school coach	(Former) player/referee
	Collegiate D3 girls’ LAX coach	Parent of LAX player(s)
Researcher (n = 3)	N/A	N/A
	N/A	N/A
	N/A	N/A
Administrator (n = 5)	National level	Parent to girls’ LAX player
	State level	N/A
	National level	N/A
	Local level	Epidemiologist/MPH
	Local level	Parent to high school athlete(s) Boys’ sports’ coach

LAX, lacrosse; MPH, master of public health; N/A, not applicable; TBI, traumatic brain injury.

**Table 3:** Themes and Subthemes

Theme	Description of theme/subthemes	Representative quotes
Playing through pain and health consequences of concussions	• Perceptions of how likely players/coaches/medical professionals are to diagnose/report/respond to a concussion • Experiences of disregarding or continuing to play through potential symptoms of a brain injury; perceptions of social pressures to play through pain • Discussion of short- and long-term effects a concussion can have on athlete health, including ongoing symptoms and treatment for both physical and mental health issues • Also can include effects on family life, future plans/education (such as college recruitment or grades in school), or having to quit the sport	“If you are in high school and you're injured sophomore year or junior year, you're not getting recruited [to play in college].” - Player
Concussion prevention strategies and the potential role of headgear	• What wearing headgear can/cannot do for a player ○ avert injury (preventative measure) ○ provide protection after an injury (reactive measure) ○ serve as a supplementary measure of protection in addition to other measures • Standardization or consistency of policies regarding protective equipment • Factors that influence the use of headgear, such as comfort, visual appeal, functionality • What role do rule enforcement, coaching style, and training of officials have on the number/severity of concussions on the field	“So having the helmet allows, it's an extra cushion. It's an extra piece of equipment that does not impede the game”-Coach “When it comes to concussions, no piece of equipment can stop the concussion. At this point that has not been proven” -Administrator
Symbolism of headgear and gender dynamics	• Influence of history, tradition, and beliefs about gender on distinctions between headgear in girl's/women's lacrosse versus helmets in boys’/men's lacrosse • What helmets represent about appropriate/expected style of play for girls and young women, including: ○ relationship between protective equipment and level of aggression/physicality in the sport, particularly comparing girls’ versus boys’ lacrosse ○ possibility of risk compensation; debates over whether protective equipment is associated with taking greater physical risks	“I think people, when they see it moving to the boys’ game, feel sad that it is just getting assimilated versus preserving its own uniqueness, right?”- Administrator “We are absolutely seeing that aesthetics is a major predictor. If it looks cool, they are more likely to wear it”-Researcher
Autonomy and decision making	• Mention of child autonomy, paternal authority, coach decision-making power, league rule making, and/or governing body action in the context of taking safety precautions to protect a player. • Discussion of governing bodies and policy-making processes used by authorities at different levels of sport (national, state, and local) • What forms of evidence are being used to inform decisions being made? • What threshold of evidence should be met before making policy changes? • Influence of player age and level of experience with & familiarity with the sport	“These people in their offices making these decisions are not really on the field. So coaches at least get a little bit closer to the field and think about player safety and opinions of players”- Coach

### Playing Through Pain and Health Consequences of Concussions

Regardless of stance on mandating headgear, a majority of participants showed concern about concussion incidence in the game and the wide-ranging effects these injuries can have on the health and trajectory of a young athlete's life. Three brain injury survivors recounted numerous lasting impacts of their concussions beyond the immediate and more obvious physical effects. These included ongoing symptoms of depression; struggles to be taken seriously as young women by male healthcare professionals; the inability to return to school; and/or otherwise circumscribed academic opportunities, including recruitment for collegiate athletics. One player shared (and several interviewees echoed) how, “a lot of male coaches, neurologists, and doctors… are definitely dismissive of females that have ‘invisible injuries’” such as concussions. This experience can lead players to either knowingly or unknowingly disregard symptoms. The problem of playing through pain is further exacerbated by multiple factors: the often limited presence of athletic trainers on the field; difficulty in recognizing signs of a concussion; a fear of how sitting out can negatively affect an athlete's future playing the game; and how players will be viewed by their teammates, coaches, and recruiters. Several interviewees described receiving praise for disregarding potential TBI symptoms from authority figures, as 1 athlete recalled, she “played through pain and my coaches were like, ‘Yeah, that's my girl.’”

### The Potential Role of Headgear and Other Concussion Prevention Strategies

Respondents expressed varying degrees of confidence in the effectiveness of headgear for the primary or secondary prevention of concussions. Some criticism and some praise were given to the functionality, comfort, and visual appeal of various headgear models. Ultimately, several participants emphasized these features rather than specific safety considerations as most strongly influencing the likelihood that a player would wear headgear, to begin with. As one researcher put it, “aesthetics is a major predictor. If it looks cool, [players] are more likely to wear it.” In addition, respondents differed over whether headgear should be viewed as the main preventative measure against concussion or as a supplementary provision to stronger enforcement of rules, but all shared the sentiment that headgear “can't be in isolation and we also need education and training and rule enforcement” to adequately protect players. A majority of interviewees strongly agreed that inconsistencies in rule implementation pose a threat to player safety, with another researcher expressing the concern that in a collision, if “it's one person wearing a helmet and the other person not wearing a helmet, the person not wearing a helmet could get pretty hurt.”

### Symbolism of Headgear and Gender Dynamics

Respondents frequently described the relationship between gender, the history of girls’ lacrosse, and how they perceived protective equipment. Headgear variously represented enhanced safety; misperceptions of athlete invincibility; a threat to maintaining tradition; or convergence to a more aggressive, masculine version of the game. One highly debated explanation for the association of helmets with a more physical style of play was risk compensation.^14^ Colloquially referred to as the gladiator effect, this concept suggests that players are more likely to take greater risks when they wear headgear and feel more protected. Some interviewees describe perceiving helmeted players as feeling invincible or playing more aggressive(ly). Meanwhile, others either felt that (1) the presence of a helmet did not alter playing style or (2) that the claim of risk compensation is a weak “outdated argument that people are still holding on to” often used to pushback against headgear despite no evidence supporting this dynamic.

These themes led to larger discussions of how historical stereotypes of athleticism for women carry over into modern debates. As one researcher explained:

You'll hear things like [the women's] game is more about technique and finesse and less about raw athleticism and power. It's like, well now you're stereotyping the [women's] game and you're making assumptions about what type of protective equipment they need based on those stereotypes.

A majority of respondents mentioned a fear that introducing helmets would threaten “preserving [the] uniqueness” of girls’ lacrosse, as one administrator put it. As described by another interviewee, this causes hesitancy toward “headgear because [many] are afraid it can manifest a change towards the men's game in a way.”

### Autonomy and Decision Making

In addition to examining if and how headgear can address the risk of concussions, interviewees addressed who should have the decision-making power. Should headgear be optional or should it be mandated? Opinions encompassed the role and influence of national governing bodies, player autonomy, and parental or coach authority. Most interviewees recognized the role of U.S. Lacrosse as a national decision-making authority in the sport. Yet, there was disagreement on whether the national governing body has an obligation to act promptly on the basis of currently available data or wait for further evidence in regard to standardizing headgear. Many respondents, primarily players, coaches, and local-level administrators, shared the sentiment that a mandate “would really need to come from USA Lacrosse or schools versus having it up to one individual person or coach.” Others felt that the decision should be made specific to geographic regions, age level, leagues, or even individual players, saying that “it should be on a state by state basis because the state knows the dynamic of their [players]” or “once you're eighteen … you can make your own choices whether you want to put that helmet on or not.”

Personal experience with the game and health literature were the most commonly mentioned sources of information shaping respondents’ judgments about headgear mandates. Numerous interviewees addressed the threshold of evidence that they felt necessary to make a sound decision; some perceived an abundance of support favoring a mandate, whereas others saw a need for further research. This opened up a conversation on how much weight should be given to those with firsthand experience and regular proximity to the game in contrast to collected research data and how these various sources of evidence influenced policy making. One local administrator described how when advocating for a headgear requirement for his school district, he “had an underestimation of the power of the story [and] overestimated how compelling the data was.”

## DISCUSSION

This study highlights the importance of examining complex social attitudes toward protective equipment in girls’ lacrosse, particularly to understand the ongoing barriers against the more widespread implementation of headgear. Previous qualitative research has examined athlete attitudes toward protective headgear in a variety of sports and contexts. Examples include mouth and face guards in Swedish ice hockey, helmets in gridiron football compared with rugby union in Canada, and the effects of helmets on tackling form in American football.^15^, ^16^, ^17^ In addition, a recent 2022 pilot study explored the attitudes toward headgear in lacrosse by surveying 25 high school players before and after 1 season of use. This study found that although players’ attitudes remained mostly unchanged after 1 season, there was a small perceived change in some behaviors during game play.^18^ This study builds on this previous research through the use of semistructured interviews to gain an in-depth understanding of the broader context shaping participants’ perspectives on headgear mandates.

Among this study's interviewees, there was shared acknowledgment that the current debate is polarizing and often viewed as a binary: either strongly in favor of or strongly opposed to headgear mandates. However, this research revealed a great amount of nuance and overlap of perspectives across all respondents, with opportunities for identifying common ground and shared values that can inform injury prevention efforts. For example, respondents consistently noted the influence of history and tradition on how girls’ lacrosse is played and ways that the introduction of headgear might change the sport. This was the case whether the change was seen as being for good (improving safety and greater standardization of equipment policies) or for worse (sacrificing the sport's integrity and potentially resulting in unintended risks). Even more broadly, the theme of gender was profoundly intertwined with how respondents discussed all other key themes. At the same time, there was an important variation in perceptions of protective gear. For example, among players interviewed, some emphasized the importance of using headgear designed specifically for girls’ lacrosse, whereas others derided such equipment as a glorified bike helmet and expressed a preference for boys’ lacrosse helmets.

Importantly, these varied perspectives come in the context of limited attention and research on safety needs in girls’ and women's sports. Existing literature reveals inadequate representation of female athletes in concussion research and minimal attention given to studying phenomena, such as the gladiator effect, specifically within the context of girls’ lacrosse.^2^^,^^19^ This means that current safety policies have been established on a foundation largely specific to research among men and sports other than girls’ lacrosse, such as hockey and football.

As is the case with many forms of protective equipment, headgear can only serve its intended purpose of protecting players against head impacts if worn willingly, properly, and consistently.^20^ For this to occur, a level of shared support and reinforcement must come from relevant adults, including the coaches, parents, and administrators of the game. Numerous respondents highlighted a lack of communication and a sense of disconnect among lacrosse stakeholders when it comes to discussing safety policies. Consequently, there is a need for greater collaboration and consensus between all relevant groups to ensure that headgear best addresses the concerns of the people who will ultimately be using it. Safety measures may be more accepted when they are not perceived as being imposed by higher authorities who are disconnected from the game on the ground.

The findings of this study compare with those of previous research on protective equipment in other sports and recreational activities in several notable ways. One similarity is exemplified by one respondent's contention that players ought to be able to decide for themselves whether or not to wear headgear after reaching age 18 years. This is consistent with a history of incorporating headgear mandates first for children before mandates for adults, as in the history of the adoption of helmets and masks in boys’ and men's ice hockey in North America, or largely only imposing mandates on children but not on adults, as in the history of bicycle helmet mandates in the U.S.^21^^,^^22^ This suggests that concerns about public health paternalism may be similarly present in girls’ and women's lacrosse. By contrast, the extent of athlete understanding of the risks of playing without protective gear may vary across contexts. For example, in 2013, Glendor found that many respondents in his study of mouth and face guards among Swedish ice hockey players lacked awareness of the consequences of dental injuries.^15^ However, in our study, many respondents expressed both awareness of the risks and firsthand experience of head injuries. In some cases, respondents reported that this increased their interest in advocacy favoring the adoption of headgear. By contrast, several respondents described having lacrosse teammates who they considered unlikely to use a helmet despite knowing the long-term effects of a concussion. These mixed findings suggest that awareness of injury risks alone is not sufficient to increase the usage of protective gear.

An important strength of this study is its qualitative nature; this analysis adds to existing quantitative research on TBI and headgear efficacy in girls’ lacrosse by including a range of in-depth perspectives from key stakeholders. The views of players and coaches are often underrepresented in sports safety research, and the inclusion of administrators at multiple levels of the sport offered insights from those directly involved with making policy decisions.

### Limitations

A limitation of this study is that the sample size was small owing to challenges associated with recruiting participants. Although the sample represented numerous roles, it did not encompass players aged <18 years, parents who did not also serve as a coach, headgear manufacturers, physicians, athletic trainers, or college coaches of teams who played with helmets. Those respondents who consented to participate largely either favored or expressed a relatively neutral or uncertain perspective on headgear, meaning that those strongly opposed to mandates were not represented. The sample also lacked diversity in regard to age, race, geographic location, and socioeconomic background. Therefore, these findings are not generalizable to the entire U.S. lacrosse community. In addition, to preserve the anonymity of participants within the small pool of respondents sampled, descriptive details on each participant were not included. This is a limitation to our study because such details can be valuable for more in-depth analysis and cross-comparison across participant perspectives. These would be valuable data to collect in a future study with a larger sample size.

## CONCLUSIONS

Future qualitative research should build on this preliminary study with a larger and more diverse sample to follow up on key themes, particularly related to equipment design, the relationship between headgear use and style of play, and policy decision-making processes. This qualitative study shows the importance of attention to the unique history of girls’ lacrosse and current narratives surrounding headgear in the sport. Deeper understanding and engagement with these social factors are crucial for injury professionals seeking to implement and sustain effective safety measures to protect athletes.
